# Substance use disorder–associated tricuspid endocarditis: Staged surgery from valvectomy to replacement

**DOI:** 10.1016/j.xjon.2026.101957

**Published:** 2026-07-10

**Authors:** Joshua G. Crane, Toyokazu Endo, Jaimin R. Trivedi, Brian L. Ganzel, Sharini Shah, Siddharth V. Pahwa, Mark S. Slaughter, Michele Gallo

**Affiliations:** Department of Cardiovascular and Thoracic Surgery, University of Louisville, Louisville, Ky

**Keywords:** tricuspid valve endocarditis, valvectomy, staged valve replacement, substance abuse

## Abstract

**Objectives:**

Optimal surgical treatment of infective tricuspid infective endocarditis (TVIE) is controversial in the setting of active substance use. This study evaluated patients who had endocarditis that was not amenable to tricuspid valve repair. In these patients, a valvectomy was performed to remove the diseased tissue. Patients were then offered a subsequent tricuspid valve replacement after proper follow-up and drug abstinence.

**Methods:**

A single-center database was queried (2015-2025) to identify adults who underwent valvectomy for TVIE not amenable to repair after failed medical therapy and persistent positive blood cultures. Patients who underwent valvectomy with documented intravenous drug abstinence for 6 months were considered for staged tricuspid valve replacement (sTVR). Patients with persistent drug use did not receive further surgical treatment. Baseline characteristics, outcomes, and survival were evaluated.

**Results:**

In total, 153 patients underwent surgical treatment for TVIE. Tricuspid valvectomy was required in 51 patients. Of the patients, 66% were female, with a median age of 30 years. There were 2 operative mortalities (3.9%). The 1-, 5-, and 8-year survival was 91%, 62%, and 51%, respectively; 26 (51%) received an sTVR and 25 (49%) did not receive sTVR (non-sTVR). In those that received an sTVR, there were 0 operative mortalities, median postoperative length of stay was 6 days, and median hours in the intensive care unit was 30. Intravenous drug use relapse after sTVR was present in 57.7% of patients. Survival at 1 and 5 years after the sTVR was 100% and 60%.

**Conclusions:**

Tricuspid valvectomy is an acceptable initial treatment in high-risk patients. Tricuspid valve replacement after completing an abstinence program from intravenous drugs for 6 months has acceptable outcomes in a complex patient population. Further study of long-term outcomes is warranted.

## Graphical Abstract

**Figure:**
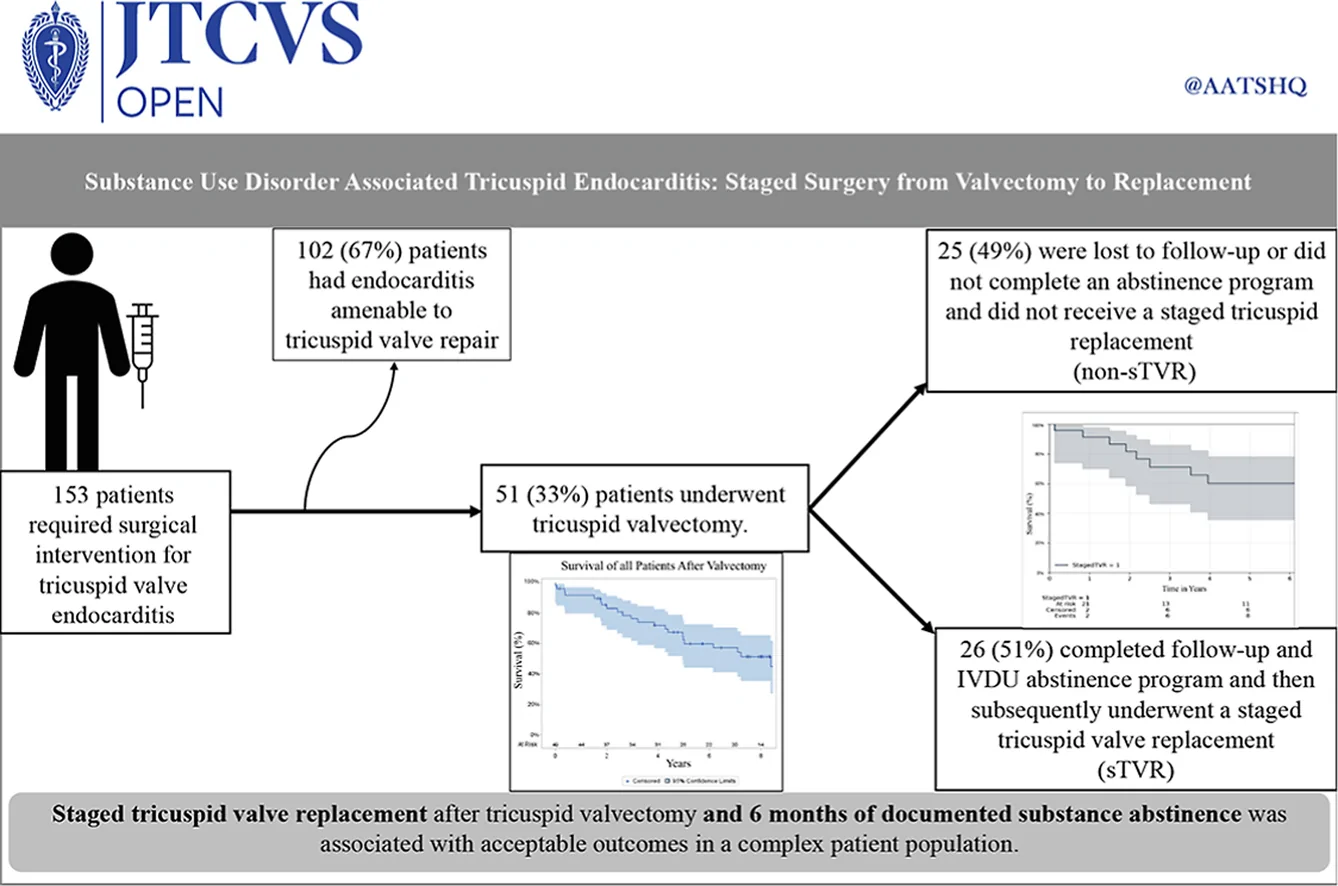

**Figure:**
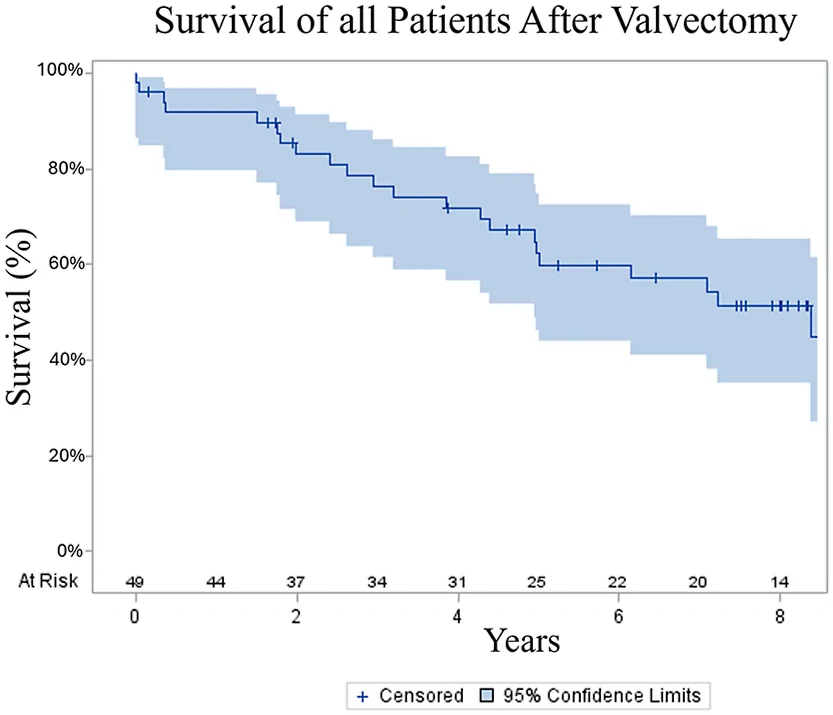
Patients with active IV drug use have acceptable long-term outcomes with a staged valve-replacement strategy.

**Central Message**

Staging tricuspid replacement in patients that are active IV drug users and not candidates for a valve repair have good long-term mortality outcomes and trends toward decreased drug relapse.

**Perspective**

Tricuspid endocarditis is frequently due to the use of contaminated needles used in substance abuse. There are concerns regarding valve replacement in active drug users who are not candidates for a valve repair due to the risk of reinfection from ongoing IV drug use. One potential option is a valvectomy with a staged valve replacement after 6 months of drug abstinence.

In the last decade, cases of infective endocarditis have sharply increased in the United States as a result of the opioid epidemic.^1^ During the COVID-19 pandemic, the economic shock and social isolation resulted in a dramatic surge in drug-overdose deaths.^2^ Contaminated drugs and inadequate sterile techniques may also cause localized and systemic infections, including cellulitis, localized abscess at the injection site, bacteremia, endocarditis, and osteomyelitis.^3^ The inpatient mortality risk of infective endocarditis is also markedly elevated, estimated at up to 10% from nationwide US databases.^1^ Although medical management with long-term intravenous (IV) antibiotics is the primary treatment strategy, several patients may not respond to treatment or have symptomatic severe tricuspid insufficiency requiring operative intervention. The optimal surgical management of tricuspid valve (TV) endocarditis aims to control/remove the source infection, usually located as vegetation on the leaflets. The American Association for Thoracic Surgery recommends TV vegetation debridement and repair of the valve when possible, although replacement is still the most commonly performed intervention.^4^ Recurrent endocarditis and subsequent prosthetic-valve endocarditis are common among IV drug users because of their greater likelihood of drug use relapse, with more than 30% of patients with infective endocarditis having repeat episodes.^5^ Reoperating on such recurrences poses a significant risk of morbidity.^6^ We assessed outcomes of patients who underwent tricuspid valvectomy for tricuspid valve infective endocarditis (TVID) in the context of intravenous drug use (IVDU). All patients with IVDU-related TVIE were offered a staged TV replacement after valvectomy and 6 months of documented IVDU abstinence. Patients were evaluated for their demographics, operative mortality, complications, and long-term mortality.

## Methods

### Study Population and Data

A single-center cardiac surgery database was reviewed between 2015 and 2025 after institutional review board approval (no. 12.0239, July 10, 2014; individual consent for publication was waived). Adult patients (aged ≥18 years) with TVIE who underwent TV repair or valvectomy were identified. Patients with a concomitant operation were also included in the final data set. Demographics, risk factors, and clinical characteristics were obtained for all patients who underwent tricuspid valvectomy.

Our approach to TVIE was standardized. In each patient with TVIE, we approached the operation with the intent to repair the valve if possible. In patients in whom the valve repair was not possible, because of the extensive nature of the TVIE, a valvectomy was performed with plans for a staged tricuspid valve replacement (sTVR) after 6 months of IVDU abstinence and reliable follow-up. Because some patients did not follow up or continue drug abstinence after their tricuspid valvectomy, they were considered “non-sTVR.”

### Analysis

The primary outcome of the study was 1-, 5-, and 8-year survival of the entire cohort after the valvectomy operation. The secondary outcomes of this study were operative mortality of the entire cohort and operative mortality of those who underwent sTVR. In addition, from those who underwent sTVR, 1- and 5-year survival, postoperative length of stay, intensive care unit length of stay, reoperation for bleeding, major stroke, prolonged ventilator use, IVDU relapse, and readmission were outcomes. Descriptive statistical methods were used to evaluate the data. Survival was evaluated with Kaplan-Meier curves using the log-rank test and a competing risk diagram. All analyses were conducted using SAS, version 9.4 software (SAS Institute Inc) at a 95% confidence interval level.

## Results

During the study period, a total of 153 patients underwent surgical treatment for TVIE secondary to substance abuse. There were 51 patients in whom the TV could not be primarily repaired, and they underwent a tricuspid valvectomy (Figure 1). Table 1 describes the demographic and preoperative risk factors among the study groups at the time of the original valvectomy. Median age was 30 years (26-33 years) and most of the patients were female (66%). In total, 3% of patients had diabetes, 9% had hypertension, median hemoglobin was 9 g/dL (8-9 g/dL), median white blood cell count was 12 cells × 10^3^/μL (9-15 × 10^3^/μL), and median creatinine was 0.8 mg/dL (0.7-1.2 mg/dL). In total, 3% of patients were on dialysis before index valvectomy, and 92% were active tobacco users. A total of 2% had aortic insufficiency of moderate or greater, and 6% had mitral regurgitation of moderate or greater. In total, 21% had moderate tricuspid regurgitation, and 54% had severe tricuspid regurgitation. Median ejection fraction was 60% (54%-63%) and 8% of the patients had previous operations. Of all patients undergoing index valvectomy, no patients were undergoing coronary artery bypass grafting or had a balloon pump. A total of 2% of patients underwent concomitant aortic valve operation, 2% underwent concomitant mitral valve operation, and 2% were on extracorporeal membrane oxygenation.

**Figure 1:**
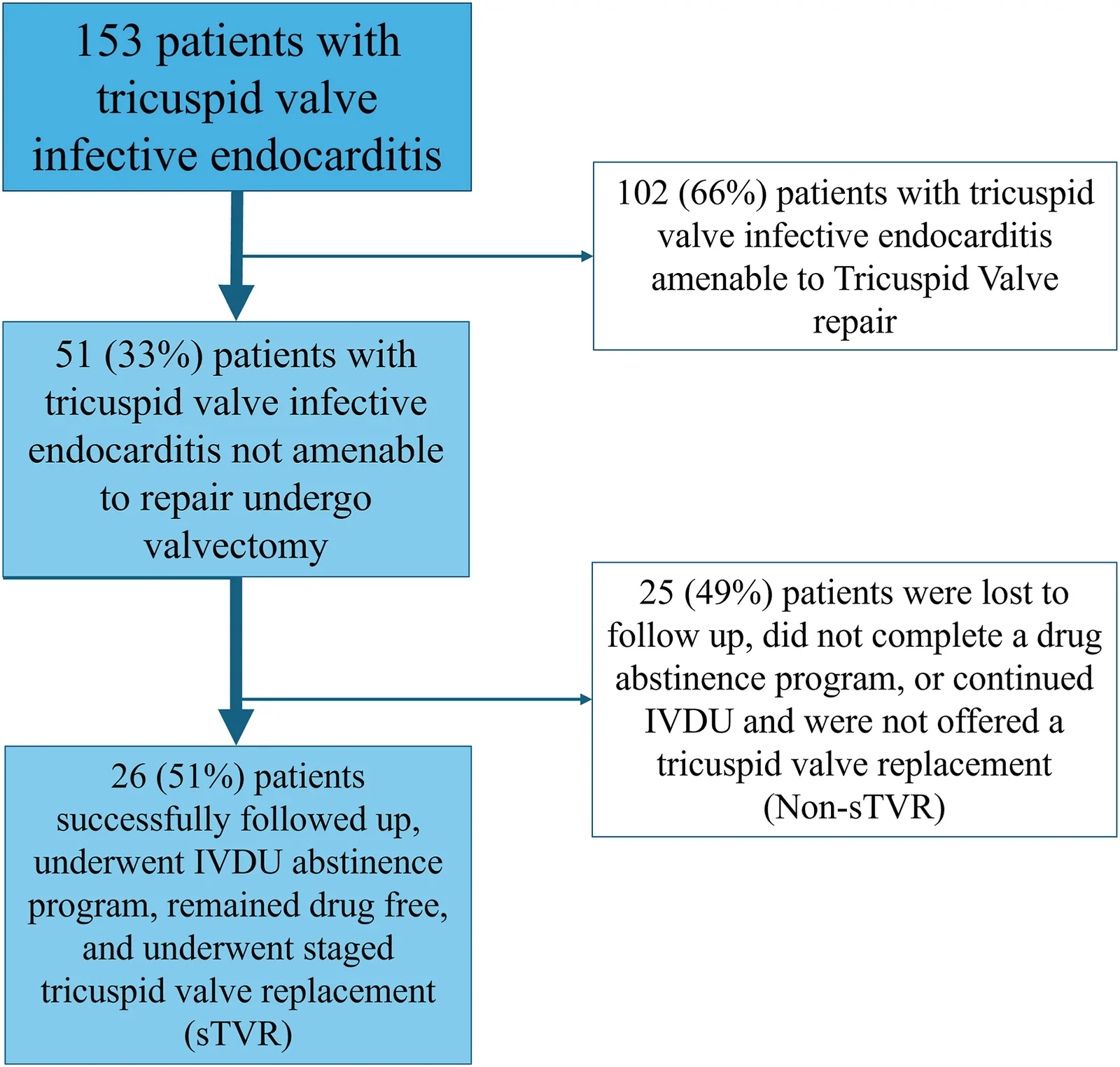
Flowchart of patient population treated in for tricuspid endocarditis. *IVDU*, Intravenous drug use; *sTVR*, staged tricuspid valve replacement.

**Table 1:** Demographics and risk factors for patients with endocarditis undergoing surgical therapy

Demographics	Valvectomy patients
Volume, n	51
Age, y	30 (26-33)
Gender, male	17 (33.3%)
Diabetes, %	2 (3.9%)
Hypertension, %	5 (9.8%)
Hemoglobin, g/dL	9 (8-9)
White blood cells, ×10^3^/μL	12 (9-15)
Creatinine, mg/dL	0.8 (0.7-1.2)
Dialysis, %	2 (3.9%)
Tobacco, %	47 (92.1%)
Aortic insufficiency greater than moderate	1 (1.9%)
Mitral regurgitation greater than moderate	3 (5.8%)
Tricuspid regurgitation	
Moderate	11 (21%)
Severe	28 (54.9%)
Left ventricular ejection fraction, %	60 (54-63)
Reoperation, %	4 (7.8%)
Concomitant procedure, %	
Coronary artery bypass graft	0
Aortic valve	1 (1.9%)
Mitral valve	1 (1.9%)
Intra-aortic balloon pump	0
Extracorporeal membrane oxygenation	1 (1.9%)

Of the 51 patients, a total of 25 (49%) patients underwent only a valvectomy and did not complete follow-up or abstinence programming (non-sTVR), and 26 (51%) patients underwent sTVR after drug abstinence programming and consistent follow-up. All patients who underwent sTVR experienced severe tricuspid regurgitation after their valvectomy (Table E1).

### Primary Outcome

Overall actuarial survival for all patients after valvectomy at 1, 5, and 8 years was 91%, 62%, and 51% (Figure 2). There were 2 operative mortalities after the index valvectomy (3.9%). Of the 25 patients classified as non-sTVR, 19 (78%) continued IVDU, and 6 (24%) patients were lost at follow-up. Mean time to follow-up was 1332 days. Eight patients classified as non-sTVR died outside of the 30-day window, with 5 deaths related to substance use overdose. Of the 26 patients classified as non-sTVR, 4 (15%) patients were lost during follow-up after their staged operation. Mean time from valvectomy to staged TV replacement was 537 days (315-856 days). Mean time to follow-up from valvectomy was 1058 days, and mean time to follow-up from staged tricuspid replacement was 521 days. One patient was readmitted with overdose and died, and 1 patient was readmitted with bioprosthetic valve endocarditis and died. Comparative risk analysis, shown in Figure 3, demonstrates the populations time from index valvectomy to death or to sTVR in order to best visualize the cohorts progress over time.

**Figure 2:**
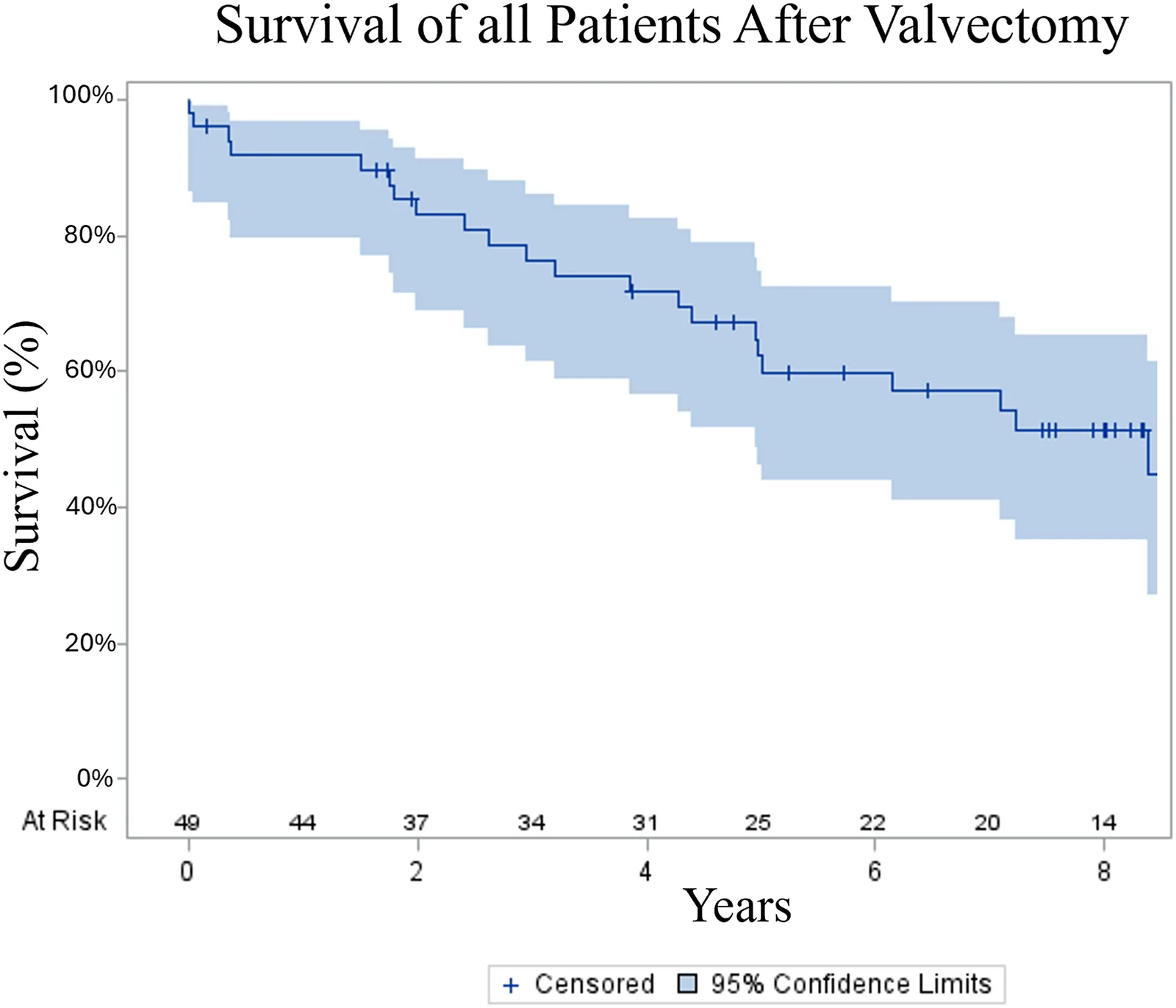
Kaplan-Meier analysis of survival from index valvectomy operation for the entire cohort. 95% CIs.

**Figure 3:**
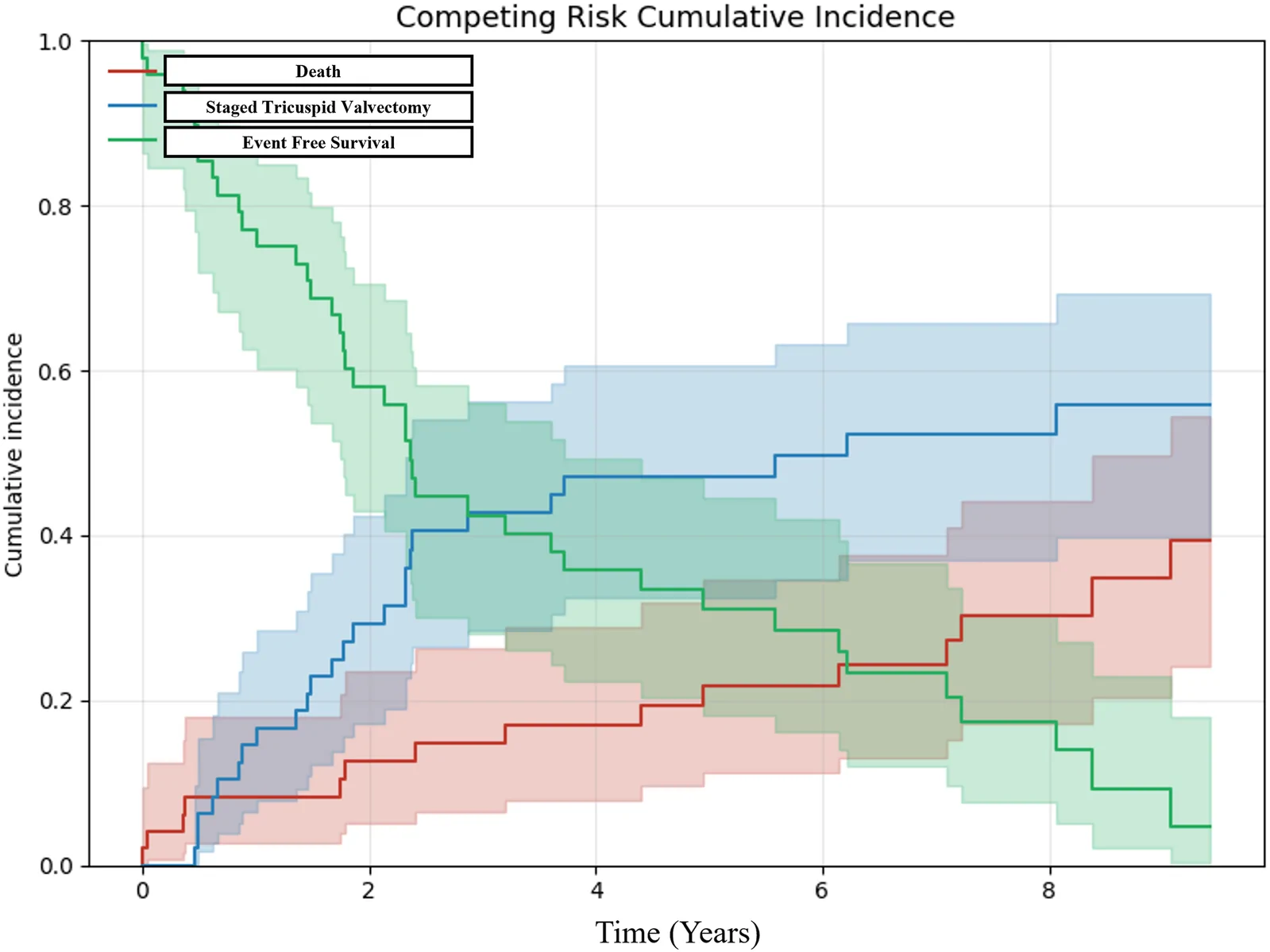
Comparative risk analysis of all patients from the time of index valvectomy. Patient outcomes of death and staged tricuspid valve replacement are compared visually.

### Secondary Outcomes

The actuarial survival after sTVR at 1 and 5 years was 100% and 60% (Figure 4). The 30-day STS operative mortality was 0% for sTVR (Table 2). After sTVR, postoperative length of stay was 6 days (5-7 days) and intensive care unit length of stay was 30 hours (26-72 hours). Reoperation for bleeding occurred in 1 (3.9%) patients. No patients had a postoperative stroke or renal failure requiring dialysis. Three patients (11.5%) had prolonged ventilator requirements. Readmission overall was seen in 20 (76.9%) patients but readmission for valve-related issues was only seen in 13 (53.8%) patients. IVDU relapse occurred in 15 (57.7%) of patients who underwent sTVR.

**Figure 4:**
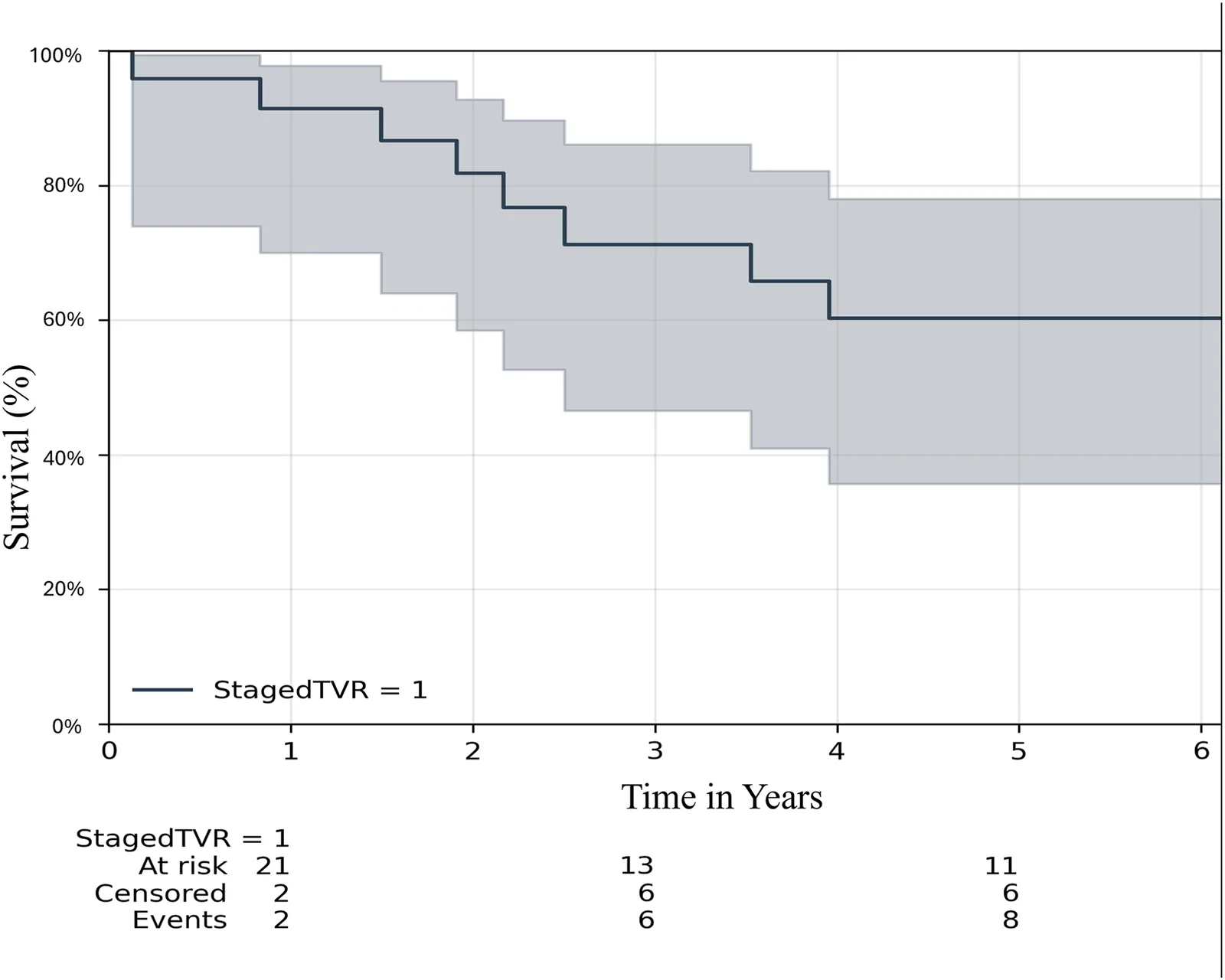
Kaplan-Meier analysis of survival from staged tricuspid valve replacement operation for the entire cohort. 95% confidence interval.

**Table 2:** Postoperative morbidity and mortality outcomes for patients undergoing staged tricuspid valve replacement for IVDU-related tricuspid valve endocarditis

Morbidity and mortality	Staged tricuspid replacement, n = 26
Total intensive care unit time, h	30 (26-72)
Postoperative length of stay, d	6 (5-7)
Operative mortality	0%
Reoperative bleeding	1 (3.9%)
Prolonged vent	3 (11.5%)
Stroke	0
Renal failure dialysis	0
Long-term outcomes	
Readmission	20 (76.9%)
Readmission valve	13 (53.8%)
IVDU relapse	15 (57.7%)

*IVDU*, Intravenous drug use.

## Discussion

This single-center analysis reports a contemporary series of patients undergoing isolated tricuspid operation for endocarditis. A staged approach is proposed, where first the TV and vegetation are removed. Valvectomy was performed when tricuspid repair was technically not feasible. Patients received the second operation (TV replacement) after documented abstinence from IV drug abuse for at least 6 months. The rationale for performing an initial tricuspid valvectomy surgery is to remove all the infected tissue and to prevent valve or prosthesis reinfection in case of active bacteriemia and or early drug use relapse. Other advantages of valvectomy include avoidance of anticoagulation therapy and lower risk of heart block necessitating a permanent pacemaker. Furthermore, the complete absence of the TV with valvectomy could prevent the recurrence of infection.

There are several important findings in our analysis. First, the procedural volume has increased over the years as a result of the opioid epidemic. Similarly, the Society of Thoracic Surgeons database revealed that the number of TV surgeries has increased by 35% between the years 2011 (n = 7814) and 2016 (n = 10,722), where the increase in the TV surgeries performed for infective endocarditis was a 500% (120 in 2011 to 628 in 2016).^7^ Historically, the use of TV replacement has been reserved for those who do not have a valve that is amenable to repair.^8^ Although valve replacement carried the same risk of operative mortality, those with replacement had a significantly increased risk of recurrent infective endocarditis.^8^ In addition, these patients had increased risk of need for reoperation, often because of valve reinfection.^8^ In one study, 21% of the patients who had undergone TV replacement for TVIE required reoperation for prosthetic TVIE after continued IVDU.^9^ In the IVDU patient population, this is unsurprising, because 30% of patients with TVIE will experience a second episode and 10% will experience a third.^5^

A disadvantage of tricuspid valvectomy is that it results in massive tricuspid regurgitation and ventricularization of right atrial pressures, which can cause significant right heart failure. Patients who present with preoperative severe pulmonary nonreversible hypertension should not undergo valvectomy. However, in patients with normal preoperative pulmonary artery pressures, the risk of right heart failure, even in long-term follow-up, is not significantly greater than those who underwent index TV replacement.^10^

IVDU relapse was noted to be 53% after the staged TV replacement. This speaks to the difficult challenges that this patient population and providers face in maintaining their health. IVDU is a complex disease that challenges patients’ physical and mental health. Despite efforts in structured programming, patients may often have IVDU relapse. Patients with primary valve replacement have increased readmission and risk for repeat endocarditis.^11^ A meta-analysis of 16 studies showed that without using a staged approach, there is no significant difference (*P* = .81) in the rate of recurrent endocarditis between valvectomy (7%) versus replacement (19%).^10^ A staged approach selects patients in terms of their ability to maintain adequate follow-up, undergo detoxification and rehabilitation, and demonstrate abstinence before TV replacement. The treatment of endocarditis without addressing the substance use disorder fails to comprehensively treat the patient and places the patient at greater risk for recurrent disease.^12^ The better outcomes in patients receiving valve-replacement surgery are likely multifactorial, suggesting the benefits of staying drug-free improve other aspects of life: healthier habits with fewer infections, improved mental and social aspects, and better integration into mainstream society.

The most critical factor in improving the survival of IV drug users with tricuspid endocarditis is treating their substance use disorder. Substance use disorder is a complex problem that requires a comprehensive treatment approach. To successfully address the issue, patients need to be educated and persuaded to cease drug use and undergo detoxification and rehabilitation treatment. The optimal treatment plan for substance use disorder involves a multifaceted approach. It should include the use of medication to reduce addiction, signed agreements for abstinence, cognitive-behavioral therapy to help patients develop coping mechanisms and manage their emotions, as well as psychosocial and financial support to help them deal with the challenges of recovery. The use of medication can be effective in reducing cravings and withdrawal symptoms. Agreements for abstinence, also known as sobriety contracts or commitment contracts, help reinforce the patient's commitment to recovery and providing a sense of accountability. Cognitive-behavioral therapy helps patients identify and modify the negative thoughts and behaviors that contribute to drug use. Finally, psychosocial and financial support can help patients address any social, financial, or legal issues that may arise during the recovery process.^13^

In recent years, as an alternative to surgery, several case series have described the use of percutaneous, vacuum-assisted devices for the removal of right-sided intracardiac masses using the AngioVac (AngioDynamics) system. George and colleagues^14^ retrospectively analyzed 33 patients with TV endocarditis who underwent successful vegetation debulking in 61% of cases using the AngioVac device. Further evaluation is warranted regarding this new technology.

This study does have limitations. The study was completed as a retrospective analysis, limiting a strong control group to determine the best course of action in IVDU with TVIE. This study shows one approach to a problem that may be approached many ways at similar institutes. However, we believe this study evaluates the safety of one single approach to a complex disease by attempting to treat it in a multifactorial way. Finally, the limitations of this population, especially in reliable follow-up, make understanding of this problem very complex.

## Conclusions

In conclusion, a staged treatment for tricuspid endocarditis with previous valvectomy and later valve replacement appears to be safe and acceptable to rehabilitate and educate patients in the setting of substance use disorder.

## Conflict of Interest Statement

The authors reported no conflicts of interest.

The *Journal* policy requires editors and reviewers to disclose conflicts of interest and to decline handling or reviewing manuscripts for which they may have a conflict of interest. The editors and reviewers of this article have no conflicts of interest.

## Figures and Tables

**Table E1:** Demographics, clinical characteristics, and operative characteristics at the time of the valvectomy for the non-sTVR group and at the time of the staged tricuspid replacement in the sTVR group

Demographics	sTVR staged tricuspid replacement, n = 26
Age, y	32 (27-34)
Gender, male	36%
Diabetes	4%
Hypertension	24%
Hemoglobin	14 (11-15)
WBC	7 (6-8)
Creatinine	0.8 (0.7-1.0)
Dialysis	0
Tobacco	96%
AI greater than moderate	0
MR greater than moderate	0
TR	
Moderate	0%
Severe	100%
EF	56% (50%-60%)
Reoperation	100%
Concomitant procedure	
CABG	0%
AV	4%
MV	0
IABP	0%
ECMO	0%

*sTVR*, Staged tricuspid valve replacement; *WBC*, white blood cell; *AI*, aortic insufficiency; *MR*, mitral regurgitation; *TR*, tricuspid regurgitation; *EF*, ejection fraction; *CABG*, coronary artery bypass grafting; *AV*, aortic valve; *MV*, mitral valve; *IABP*, intra-aortic balloon pump; *ECMO*, extracorporeal membrane oxygenation.
